# Complete Genome Sequence of Porcine Circovirus Type 2 Strain HB-MC1

**DOI:** 10.1128/genomeA.00987-14

**Published:** 2014-12-24

**Authors:** Wanzhe Yuan, Jiefeng Li, Yi Zuo, Jiguo Sun, Chunfu Xie, Xiaoyuan Wei, Qiuliang Zhang, Guohua Yan, Yuzeng Yang, Qingsuo Li

**Affiliations:** a College of Animal Medicine, Agricultural University of Hebei, Baoding, Hebei, China; b Hebei Engineering and Technology Research Center of Veterinary Biological Products, Baoding, Hebei, China; c North China Research Center of Animal Epidemic Pathogen Biology, China Agriculture Ministry, Baoding, Hebei, China; d Hebei Institute of Animal Husbandry and Veterinary Science, Baoding, Hebei, China

## Abstract

Porcine circovirus type 2 (PCV2) is the primary causative agent of porcine circovirus-associated disease (PCVAD). Here, we report the complete genome sequence of PCV2 strain HB-MC1, which belongs to PCV2d.

## GENOME ANNOUNCEMENT

Porcine circovirus type 2 (PCV2) is associated with distinct syndromes and diseases in swine, collectively known as porcine circovirus-associated diseases (PCVAD), which include postweaning multisystemic wasting syndrome (PMWS), PCV2-associated pneumonia as a part of the porcine respiratory disease complex (PRDC), PCV2-associated enteritis, PCV2-associated reproductive failure, and porcine dermatitis and nephropathy syndrome (PDNS) (1–3). PCV2-infection is widespread and essentially all pig herds are infected with PCV2.

Porcine circovirus 2 (PCV2), a member of the genus *Circovirus* in the family *Circoviridae*, is a very small single-stranded negative-sense DNA virus of approximately 1.7 kb (4). The genome of PCV2 encodes three major open reading frames (ORFs) encoding the replicase proteins (ORF1), the viral capsid protein (ORF2), and a protein with suggested apoptotic activity (ORF3) (5). Previous reports showed that there were five PCV2 genotypes, including PCV2a, PCV2b, PCV2c, PCV2d, and PCV2e (6–9). Here, we report the complete genome sequence of a novel PCV2d strain, HB-MC1.

HB-MC1 was isolated from tissue samples from pigs with PMWS in a commercial pig farm in Hebei province in 2014. The full-length genome of the isolated strain was amplified by using PCV2-specific primers. The PCR products were purified and cloned into the pEASY-Blunt cloning vector (Transgen, China), and subsequently subjected to automated sequencing reaction by the Invitrogen company (Invitrogen, China). Genomic analyses were conducted using DNAMAN (University of California). Phylogenetic trees were constructed using MEGA5. The complete genomic sequence of HB-MC1 was determined to be 1,767 nucleotides in length, with a GC content of 48.5%. It consists of at least three ORFs, encoding 2 major proteins, the Rep and cap proteins. Multiple sequence alignment based on the ORF2 was completed by means of DNAStar software (DNAStar, Madison, WI, USA) with other available strains from the GenBank nucleotide database. HB-MC1 shares a high identity (98.2% to 99.9%) with the strains TJ, NIVS-C, and SD. The phylogenetic tree based on the ORF2 nucleotide sequences showed that HB-MC1 isolates belong to genotype 2d. The relationship between the genomic information and pathogenicity needs to be further investigated.

### Nucleotide sequence accession number.

The genome sequence of PCV2 strain HB-MC1 has been deposited in GenBank under the accession number KM460824.
